# Socio-economic inequalities in the association between alcohol use disorder and depressive disorder among Thai adults: a population-based study

**DOI:** 10.1186/s12888-020-02958-6

**Published:** 2020-11-23

**Authors:** Sawitri Assanangkornchai, Jiraluck Nontarak, Wichai Aekplakorn, Suwat Chariyalertsak, Pattapong Kessomboon, Surasak Taneepanichskul

**Affiliations:** 1 Department of Epidemiology, Faculty of Medicine, Prince of Songkla University, Hat Yai, Songkhla, 90110 Thailand; 2 Department of Epidemiology, Faculty of Public Health, Mahidol University, Bangkok, Thailand; 3 Department of Community Medicine, Faculty of Medicine Ramathibodi Hospital, Mahidol University, Bangkok, Thailand; 4 Faculty of Public Health, Chiang Mai University, Chiang Mai, Thailand; 5 Faculty of Medicine, Khon Kaen University, Khon Kaen, Thailand; 6 Faculty of Medicine, Chulalongkorn University, Bangkok, Thailand

**Keywords:** Alcohol use disorder, Depressive disorder, Socio-economic status, wealth index, National survey

## Abstract

**Background:**

Previous evidence indicates significant associations between depressive disorders and alcohol use disorder (AUD) and their strong links with social conditions. This study aims to investigate the association between major depressive episode (MDE) and AUD across various socio-economic groups.

**Methods:**

We analysed data from the 2014 Thai National Health Examination Survey containing a random sample of 13,177 adults aged > 20 years from the general population. The Alcohol Use Disorder Identification Test was used to classify respondents into non-problem drinking (score 0–7), hazardous drinking (score 8–15), and harmful-dependent drinking (score 16–40). MDE was identified using questions based on the DSM-IV. Adjusted odds ratios (AOR) and 95% confidence intervals (CI) were calculated using multinomial logistic regression to determine the strength of associations between MDE as a predictor and AUD as an outcome variable across different socio-economic levels.

**Results:**

The prevalence of MDE, hazardous, and harmful-dependent drinking was 2.5, 10.3, and 1.9%, respectively. The association between MDE and AUD was modified by wealth index, education level and area of residence. AORs for the association between MDE and harmful-dependent drinking were high among those in the highest (AOR = 8.68, 95% CI: 5.34, 14.11) and lowest (AOR = 7.14, 95% CI: 3.71, 13.73) levels of wealth index but not significant among those in the middle level (AOR = 1.78, 95% CI: 0.74, 4.25). Education had the strongest effect on the relationship between MDE and harmful-dependent drinking (AOR = 16.0, 95% CI: 10.30, 24.90 among those completing secondary school or higher and AOR = 1.44, 95% CI: 0.63, 3.33 among those completing primary school only). The association between MDE and harmful-dependent drinking was higher among people who lived in urban areas (AOR = 8.50, 95% CI: 5.50, 13.13) compared to those living in rural areas (AOR = 4.73, 95% CI: 3.31, 6.77).

**Conclusion:**

Socio-economic factors modify the association between alcohol use disorder and major depressive disorder among Thai people.

## Background

Alcohol use disorder (AUD) and depressive disorders are significant public health problems worldwide and both are strongly linked to social conditions [1, 2]. These two disorders are among the most prevalent psychiatric disorders and often co-occur. The co-existence of these disorders increases disability and worsens the prognosis [3–8]. Research evidence reveals some potential developmental pathways for the co-occurrence of AUD and depressive disorders, including: (1) depressive disorders increase the risk for AUD, (2) AUD increases the risk for depressive disorders, and (3) both conditions share a similar pathophysiology or have common risk factors, yet these shared risks remain poorly understood [8].

There is a known relationship between socio-economic status (SES) and mental health [9–16]. Among the social problems of concern is inequality. In terms of alcohol use disorder, people with a relatively low SES may consume similar amounts of alcohol to those with a relatively high SES, but those with a lower SES may have a higher-than-expected burden of negative consequences of alcohol consumption [17]. However, those with alcohol use disorder may suffer from loss of employment and income and eventual reduction in SES, thus the reverse causality cannot be precluded in the interpretation of the relationship. A study from the United States found that people with a relatively lower SES had a higher risk of co-occurring mental health and alcohol-related problems, whereas people with greater experiences of alcohol-related problems tended to have lower incomes [18]. With regard to depression, people with a lower SES tend to have a higher odds of being depressed than people with a higher SES, although the presence and strength of this association varies according to contextual factors such as geographical region and historical period [19].

The socio-economic inequalities in mental disorders, including alcohol use disorder and depressive disorders, may be explained by causation and selection processes. Individuals in a lower SES group may experience mental health disorders (causation) because of the high exposure to unfavorable material, psychosocial, and behavioral factors. In contrast, preexisting psychiatric problems impair ones’ ability to attain or retain a higher SES status, leading to a downward SES (selection) [10].

Geographic location, i.e. rural or urban, has been shown to be associated with discrete culture and social environment that may impact people’s behaviors and health condition [20, 21]. In Thailand, while urban residents have more access to government-sponsored public services, e.g. jobs, education, and health care, rural residents have limited access to these community resources and opportunities. One of the Sustainable Development Goals is to reduce inequality within and among countries by empowering and promoting the social, economic and political inclusion of all people, irrespective of their age, sex, disability, race, ethnicity, origin, religion, economic situation or any other factors [22]. In order to identify the underlying drivers of health outcomes so that efforts can be made to reduce inequalities, researchers are increasingly examining social determinants of health [11, 12]. Social inequality in mental health occurs in rich and poor countries in different ways. Understanding the situation in Thailand, an upper middle-income country with different sociocultural background from most previous studies, would be an important achievement.

Although there have been several studies investigating the association between depressive disorder and AUD, and between each of these disorders and SES as aforementioned, there is less research on how the association between depressive disorder and AUD may vary across different levels of SES. As all previous literature indicates disparities in the risk of depressive disorder and AUD among people with different levels of SES, it is plausible that the causal link between depressive disorder and AUD might also differ across SES, which may be explained by different capacities between the better-off and disadvantaged depressed individuals in coping with and seeking support for their depressive condition. Based on our literature search, we have found only one study examining the associations between depressive symptoms and alcohol use across socio-economic levels [23], which was conducted in 40- and 45-year-old Norwegian adults. Thus, there is a need for more research investigating the modifying effect of SES on the relationship between depressive disorder and AUD, which would add to our understanding on social inequalities of mental health disorders, including the most common co-occurring disorders, i.e. alcohol use disorder and depressive disorder.

This study aimed to examine the past 12-month prevalence of major depressive episode (MDE) and alcohol use disorder (AUD) and the association between these two conditions at different socio-economic levels among a population-based sample aged 20 and over in Thailand. Our conceptual framework for this study is that people with MDE would have an increased likelihood for AUD and this association might be modified by socio-economic status variables, including wealth index, education, employment status and area of residence while other factors such as age, sex, marital status and the presence of chronic disease might confound such an association.

## Methods

### Design and setting

This study used data from Thailand’s fifth National Health Examination Survey (NHES-5), which was conducted in 2014. Surveys in this series have been conducted approximately every *5* years since 1991. The surveys are designed to represent non-institutionalized Thai populations, using a multistage stratified sampling method, the design of which has been described elsewhere [24]. Only data from adults (age 20 years and over, N = 13,177) within 540 electoral areas of 20 provinces were used in this study. Data on demographic characteristics, alcohol use patterns and disorders, and symptoms of a major depressive episode were collected by face-to-face interview by well-trained field workers, who were at least bachelor degree graduates. Anonymity of the data was assured to the participants after they were given detailed information about the study procedures and before they signed the informed consent form.

### Measures

#### Alcohol use disorder

Alcohol use disorder (AUD) was identified based on the 10-item Alcohol Use Disorder Identification Test (AUDIT), a screening instrument developed by the World Health Organization (WHO), which includes questions on the patterns of hazardous and harmful alcohol use and dependence symptoms in the past 12 months [25]. The respondents were classified into three levels based on the AUDIT scores: non-problem drinkers (0–7), hazardous drinkers (8–15), and harmful-dependent drinkers (16–40) [25].

#### Major depressive episode

Questions on major depressive episode (MDE) were derived from the Mini-International Neuropsychiatric Interview (MINI), Thai version 5.0.0-Revised 2007 [26]. The interview started with three screening questions asking if the respondent ever had depressed moods, loss of interest, and/or loss of energy or constant feelings of tiredness lasting more than a few days in the past 12 months. Those who responded in the affirmative to either one of these three questions were asked a further set of 12 questions: the first one asking if the feelings of sadness, boredom, loss of interest, and/or loss of energy occurred every day, or almost every day, and the second if those symptoms occurred for at least 2 weeks. Other questions concerned associated symptoms such as loss of appetite; psychomotor retardation; feelings of guilt or hopelessness, and thoughts of death or suicidal attempt. Also included in the questionnaire were questions asking if the respondent had ever been diagnosed with a depressive disorder or been prescribed treatment for depressive disorder in the past 12 months. The presence of MDE in this study was defined according to the Diagnostic and Statistical Manual of Mental Disorders, Fourth Edition (DSM-IV) criteria for major depressive episode as having positive answers to the first two questions and at least four associated symptoms drawn from the positive answers to the next 10 questions, or a positive answer to either of two questions on being diagnosed as or prescribed treatment for depressive disorder. The questionnaire was developed by the NHES Development Team for use in the previous fourth survey of the series [27].

#### Socio-economic status

A wealth index based on household assets using the method suggested in the MEASURE DHS+ surveys [28] was used to measure socio-economic status. The preferable use of a wealth index as a relative index of economic status is because of the lower volatility of wealth as compared with that of income and expenditures. Using a structured questionnaire, respondents were asked whether or not they or their household owned any of the following assets: a bed, air conditioner, electric water boiler, washing machine, microwave, personal computer, house telephone, car, and flushing toilet. The wealth index was calculated based on ownership of these household items. Principal components analysis was used to assign the indicator weights by first standardizing the indicator variables (calculating z-scores), then calculating factor loadings. Finally, for each household, the indicator values were multiplied by the loadings and summed to produce the household’s index value, which itself was a standardized score with a mean of zero and a standard deviation of one [28]. The index was classified into three terciles based on the distribution of the household population where the first tercile represented the lowest (poorest) SES group and the third tercile the highest (most well-to-do) SES group. Highest education attainment, employment status (yes/no) and area of residence (urban or rural) were also used as indicators of socio-economic status. Education level was categorized into primary school or lower and secondary school or above.

#### Other variables

Other potential confounding or effect modifying variables included in the analysis were demographic factors such as age, sex and marital status, as well as smoking status and presence of chronic diseases such as diabetes mellitus, hypertension, cholesterolemia, cardiovascular diseases, stroke, and cancer. Diagnoses of diabetes mellitus, cholesterolemia, and hypertension was based on the results of fasting blood sugar and cholesterol levels and blood pressure level, respectively taken at the time of data collection, or a self-report of having been diagnosed by a medical professional as having that medical condition, or being currently on pharmacological treatment for that condition. However, the presence of cardiovascular diseases, stroke, cancers, or other chronic diseases was based on self-report only.

### Statistical analysis

All analyses were weighted to take into account the different probabilities of a respondent being selected in the sample. Prevalence was expressed as a percentage. As our objective was to assess the modifying effects of SES and area of residence on the association between MDE and AUD, we first cross-tabulated MDE and AUD across levels of SES and area of residence variables. Then we examined the associations between MDE and either hazardous drinking or harmful-dependent drinking using univariate analysis. We then used multinomial logistic regression to test association between MDE and these variables with AUD. In all models, AUD was the dependent variable with three categories; non-problem drinking (reference category), hazardous drinking, and harmful-dependent drinking while MDE (yes/no) was the main independent variable. Potential confounding variables that we assessed in the analyses included age, sex, marital status, religion, smoking status, and presence of a chronic illness. Next, we assessed effect modifications by wealth index, education, employment, and area of residence by including interaction terms in the models. Wald’s chi-square tests were used to evaluate the significance of these interactions.

Lastly, to observe whether the association between MDE and AUD varied by wealth index, education, employment, and area of residence, we then stratified our analyses by levels of the potential effect modifiers whose interaction terms were statistically significant. Adjusted odds ratios (AOR) and 95% confidence intervals (CI) were presented to assess the strength of the associations. Statistical significance was evaluated at the 0.05 level and all tests were two-sided.

## Results

### Participant characteristics

Altogether, 13,177 participants (51.8% female) were included in the analysis. The mean age of the participants was 46.7 years (SD: 15.7) and 70.5% were married or in a de facto relationship. Of all participants, 57.8% had no formal education or had attained only a primary school level of education, 55.6% were employed in labor type work while 34.1% were employed in private or government work, and 10.3% were unemployed; 56.5% were living in rural areas.

Based on the AUDIT, 10.3% (95% CI: 9.8, 10.8) and 1.9% (95% CI: 1.7, 2.1) were classified into hazardous drinkers and harmful-dependent drinkers, respectively while 2.5% (95% CI: 2.3, 2.7) met the criteria for MDE in the past 12 months before the survey. Approximately 20% (95% CI: 18.9, 20.3) were current smokers. Chronic diseases were found among 41.7% (95% CI: 41.2, 43.0) of participants, with 10.2% (95% CI: 9.6, 10.7) having diabetes mellitus, 29.2% (95% CI: 28.1, 30.4) hypertension, 11.6% (95% CI: 10.4, 12.7) cholesterolemia, 3.3% (95% CI: 3.0, 3.7) cardiovascular diseases and stroke, 0.8% (95% CI: 0.7, 0.9) cancers and 15.3% (95% CI: 14.7, 15.8) other diseases.

### Prevalence of MDE and AUD by socio-economic status and area of residence

The prevalence of MDE varied by all three socio-economic status indicators but not by area of residence. The prevalence of MDE was lower in the groups with higher levels of wealth, education, and among those who were employed (Table 1).

**Table 1:** Weighted prevalence (%, (95% Confidence Interval)) of major depressive episode and alcohol use disorder in the past 12 months by wealth index, education, employment status, and area of residence (*N* = 13,177)

Category	Total (N = 13,177)	Wealth index			Education		Employment status		Area of residence	
		Tercile 1 (*N* = 4196)	Tercile 2 (*N* = 4017)	Tercile 3 (*N* = 4964)	1′ school (*N* = 8608)	2′ school (*N* = 4434)	Employed (*N* = 7888)	Unemployed (*N* = 866)	Urban (*N* = 7068)	Rural (*N* = 6109)
MDE (*N* = 406)	2.5 (2.3, 2.7)	3.1 (2.8,3.5)	2.4 (2.1,2.8)	1.9 (1.6,2.2)	3.1 (2.9,3.4)	1.7 (1.5,2.0)	2.1 (1.9,2.3)	2.7 (2.1,3.5)	2.6 (2.3,2.9)	2.4 (2.2,2.7)
		*P*-value = < 0.001			*P*-value = < 0.001		*P*-value = 0.047		P-value = 0.124	
Non-problem (*N* = 11,983)	87.8 (87.4,88.3)	85.8 (84.9,86.7)	87.8 (87.1,88.5)	90.0 (89.1,90.6)	89.6 (89.1,90.0)	85.4 (84.8, 86.0)	85.6 (85.0,86.2)	88.5 (86.4,90.3)	88.4 (87.8,89.0)	87.3 (86.8,87.9)
Hazardous (*N* = 1019)	10.3 (9.8,10.8)	11.3 (10.4,12.2)	11.0 (10.1,11.8)	8.7 (7.9,9.5)	8.9 (8.3,9.4)	12.3 (11.5,13.2)	12.2 (11.5,12.8)	9.9 (8.4,11.7)	9.5 (8.9,10.2)	10.9 (10.2,11.6)
Harmful (N = 175)	1.9 (1.7,2.1)	2.9 (2.5,3.5)	1.2 (1.0,1.5)	1.4 (1.2,1.8)	1.6 (1.4,1.8)	2.3 (2.0,2.6)	2.3 (2.0,2.5)	1.6 (1.1,2.3)	2.1 (1.8,2.4)	1.8 (1.5,2.0)
		*P*-value = < 0.001			*P*-value = < 0.001		*P*-value = 0.117		*P*-value = < 0.001	

*P*-values from Chi-squared test, *1′ school* primary school, *2′ school* secondary school or higher. Wealth index: *Tercile 1* lowest (poorest) and *Tercile 3* highest (wealthiest)

AUD was differentially distributed in different levels of wealth index, education levels, and area of residence. The highest prevalence of harmful-dependent drinking was found among people in the lowest wealth tercile and those with a secondary or higher level of education (12-year schooling). The prevalence of hazardous drinking was significantly higher among those living in rural areas but there was a non-significant difference in the prevalence of harmful drinking between those in urban and rural areas. Furthermore, no significant difference in AUD prevalence was found for employment status (Table 1).

### Association between MDE and AUD across levels of wealth index, education and area of residence

Table 2 shows crude odds ratios for the relationship between MDE and each of the SES variables with AUD. MDE was significantly associated with AUD. The prevalence of MDE among non-drinkers, hazardous drinkers, and harmful drinkers was 2.6% (standard error; SE = 0.01), 1.3% (SE = 0.10) and 6.3% (SE = 0.9), respectively. Other variables significantly associated with AUD were wealth index, education, and area of residence while employment status was marginally significant.

**Table 2:** Crude odds ratios and 95% confidence intervals for the associations between major depressive episode, socio-economic status variables and area of residence with alcohol use disorder

Characteristic	Hazardous drinking (***N*** = 1019)	Harmful-dependent drinking (***N*** = 175)	***P***-value
**Major depressive episode**			
No	1	1	< 0.001
Yes	0.52 (0.43, 0.63)	2.55 (1.80, 3.62)	
**Wealth index**			
Tercile 3 (wealthiest)	1	1	< 0.001
Tercile 1 (poorest)	1.36 (1.23, 1.50)	2.11 (1.74, 2.58)	
Tercile 2	1.29 (1.17, 1.43)	0.86 (0.62, 1.43)	
**Education**			
2′ school	1	1	< 0.001
1′ school	0.69 (0.66, 0.72)	0.66 (0.57, 0.77)	
**Employment status**			
Employed	1	1	0.05
Unemployed	0.79 (0.65, 0.96)	0.68 (0.47, 0.98)	
**Area of residence**			
Urban	1	1	< 0.001
Rural	1.16 (1.09, 1.24)	0.87 (0.77, 0.98)	

Significant interactions between MDE and wealth index (coefficient: 0.05, 95% CI: − 0.00, 0.10 for tercile 2 and coefficient: 0.08, 95% CI: 0.04, 0.12 for tercile 3), education (coefficient: 0.49, 95% CI: 0.02, 0.80) and area of residence (coefficient: -0.06, 95% CI: − 0.11, − 0.01) were found, indicating that these factors modified the association between MDE and AUD. However, no significant interaction between MDE and employment status (coefficient: -0.02, 95% CI: − 0.08, 0.03) in the association with AUD was seen.

Table 3 shows weighted percentages of AUD among those with and without MDE and adjusted odds ratios for the association between MDE and AUD across various socioeconomic levels. Adjusted for other variables, the associations between MDE and either hazardous or harmful-dependent drinking were strongest among those in the third tercile of wealth index (AOR = 2.23, 95% CI: 1.51, 2.72 for hazardous drinking and AOR = 8.68, 95% CI: 5.34, 14.11 for harmful-dependent drinking). The AOR for the association between MDE and harmful-dependent drinking was also significant among those in the first tercile of wealth index (AOR = 7.14, 95% CI: 3.71, 13.73) but lower and not significant among those in the second tercile (AOR = 1.78, 95% CI: 0.74, 4.25). The association between MDE and either hazardous or harmful-dependent drinking was significant in people who had a secondary school level of education or above (AOR = 1.75, 95% CI: 1.33, 2.30 for hazardous drinking and AOR = 16.0, 95% CI: 10.3, 24.9 for harmful-dependent drinking) but not among those with a primary school level of education or lower, indicating a strong influence of educational level on the association. Finally, the association between MDE and harmful-dependent drinking was stronger among those living in urban areas (AOR = 8.5, 95% CI: 5.50, 13.13) than in rural areas (AOR = 4.7, 95% CI: 3.31, 6.77). Regarding employment status, there were significant associations between MDE and both hazardous (AOR = 1.26, 95% CI: 1.02, 1.56) and harmful-dependent drinking (AOR = 6.81, 95% CI: 4.71, 9.85) among those who were employed but the number of unemployed participants with MDE was too small for conducting a meaningful stratified analysis.

**Table 3:** Weighted prevalence of alcohol use disorder among those with and without major depressive episode and adjusted odds ratios for the association between major depressive episode and alcohol use disorder across levels of wealth index, education, employment status, and area of residence among the Thai adult population (*N* = 13,177)

Effect modifying variable		Depressive episode	Alcohol use disorder					
			Non-problem drinking		Hazardous drinking		Harmful-dependent drinking	
Variable	Level	Status	% (95% CI)	AOR	% (95% CI)	AOR (95% CI)	% (95% CI)	AOR (95% CI)
**Wealth index**	Tercile 3	No	90.0 (89.2,90.7)	1	8.7 (8.1,9.3)	1	1.4 (1.2,1.6)	1
		Yes	85.9 (81.6,89.3)		8.4 (6.1,11.4)	2.23 (1.51,2.72)	5.8 (3.7,8.8)	8.68 (5.34,14.11)
	Tercile 2	No	87.7 (87.0,88.4)	1	11.1 (10.5,11.6)	1	1.2 (1.0,1.5)	1
		Yes	92.6 (89.1,95.0)		6.6 (4.5,9.7)	1.53 (1.02, 2.31)	0.8 (0.4,1.7)	1.78 (0.74,4.25)
	Tercile 1	No	85.7 (84.8,86.6)	1	11.5 (10.8,12.3)	1	2.8 (2.5,3.1)	1
		Yes	90.0 (85.1,93.4)		3.1 (2.3,4.2)	0.69 (0.44,1.07)	6.9 (4.0,11.6)	7.14 (3.71,13.73)
**Education**	Secondary school or higher	No	85.6 (84.9,86.2)	1	12.4 (11.8,12.9)	1	2.1 (1.9,2.4)	1
		Yes	76.7 (72.3,80.6)		9.1 (6.9,12.0)	1.75 (1.33, 2.30)	14.2 (10.4,19.1)	16.0 (10.3, 24.9)
	Primary school	No	89.4 (88.9,89.9)	1	9.0 (8.6,9.4)	1	1.6 (1.5,1.8)	1
		Yes	94.9 (93.6,95.9)		4.1 (3.4,5.0)	1.04 (0.82, 1.31)	1.0 (0.5,2.0)	1.44 (0.63, 3.33)
**Employment status**	Employed	No	85.7 (85.0,86.3)	1	12.2 (11.7,12.7)	1	2.1 (2.0,2.3)	1
		Yes	82.9 (79.5,85.7)		8.9 (7.3,10.8)	1.26 (1.02, 1.56)	8.3 (6.0,11.3)	6.81 (4.71, 9.85)
	Unemployed	No	88.3 (86.2,90.2)	1	10.0 (8.4,11.9)	1	1.6 (1.2,2.3)	1
		Yes	94.7 (91.9,96.6)		5.3 (3.4,8.2)	undetermined	0	undetermined
**Area of residence**	Urban	No	88.5 (87.9,89.1)	1	9.6 (9.2,10.1)	1	1.9 (1.7,2.1)	1
		Yes	86.9 (84.9,88.6)		5.9 (4.5,7.5)	1.25 (1.05, 1.50)	7.3 (5.2,10.3)	8.50 (5.50, 13.13)
	Rural	No	87.2 (86.6,87.8)	1	11.0 (10.6,11.6)	1	1.7 (1.6,1.9)	1
		Yes	92.2 (89.7,94.1)		5.2 (4.1,6.7)	1.24 (0.90, 1.71)	2.6 (1.7,4.0)	4.73 (3.31, 6.77)

Reference category for outcome = non-drinking or non-problem drinking based on Alcohol Use Disorder Identification Test (AUDIT) score: 0–7, hazardous drinkers = AUDIT score: 8–15 and harmful-dependent drinkers = AUDIT score: 16–40

Reference level for exposure = no major depressive episode

Wealth index: Tercile 1 = lowest (poorest) and Tercile 3 = highest socio-economic group

*AOR* adjusted odds ratio, *95% CI* 95% confidence interval, all percentages are weighted for stratified sampling survey

All odds ratios are adjusted for other socio-economic status variables, area of residence, age group, sex, marital status, religion, smoking status and presence of chronic illness

## Discussion

### Main findings, interpretations, and comparisons with previous studies

In this study, data from a large national survey among adults were used to examine the association between MDE and AUD and the effects of socio-economic factors on this association. In agreement with several studies [7, 23, 29–33] our study found a significant association between depressive disorder and AUD. However, our results do not provide support for the evidence of a stronger association between MDE and AUD among people with lower SES, but rather indicate that the SES impact on mental disorders is controversial which may vary between countries [19] and the types of measurements used for either depressive and alcohol use disorders or SES variables [23]. The significant relationship between MDE and AUD could be explained in terms of shared common genetic and environmental factors in the comorbidity of depressive disorder and AUD, as investigated in other studies [34–36]. It could also be explained in terms of causality where depressed persons may turn to alcohol as self-medication for their symptoms and develop AUD afterwards [37, 38]. However, confirming this explanation is beyond the scope of this study.

The prevalence of MDE was higher among harmful-dependent drinkers and non-drinkers than that in hazardous drinkers, making those with MDE seeming less likely to have hazardous drinking, compared to those with non-drinking behaviors. This should not be simply interpreted as that MDE was a protective factor against hazardous drinking because such an association was not controlled for other confounding factors. In the multivariate analyses when we controlled for confounding factors and stratified by level of SES variables, we found that most of the associations between MDE and hazardous drinking were not significant, and among the significant ones, increased likelihoods of MDE on hazardous drinking were found. Nevertheless, if we explain our findings on the relationship between MDE and AUD in terms of a causal pathway, it could be argued that if depressed individuals use alcohol for self-medication of their depressive symptoms, which are usually chronic, then they may use it in a harmful pattern and be more likely to become harmful or dependent drinkers than hazardous drinkers. This may therefore explain the higher prevalence of MDE among harmful-dependent drinkers than that among hazardous drinkers.

In agreement with other studies [23], the association between MDE and AUD, in particular harmful-dependent drinking, varied across different levels of socio-economic status. We observed significant associations between MDE with harmful-dependent drinking at high and low terciles of wealth index but not in the middle tercile. We also found a significant association between MDE and either hazardous or harmful-dependent drinking in those with a secondary school or higher level of education but not in those with a primary school level of education, and a stronger association among those living in urban areas compared to those living in rural areas. Inequalities in the prevalence of MDE and AUD between levels of wealth index, education, employment, and area of residence were also found, which supports studies investigating social inequalities in mental health conditions within a country [11, 13, 19, 39].

The reason that the strengths of association between MDE and harmful-dependent drinking among those in the highest and lowest socio-economic groups were significant and higher than that in the middle group is unclear. On the one hand, it could be explained by the fact that people in the high SES group may have more means to access alcohol or other drugs for self-medication or as a coping strategy when they get depressed. Major depression is a chronic illness and cognitive deficits are frequently observed in those suffering from major depression even at the first episode [40] which can in turn affect the individuals’ coping style. Those who are suffering from symptoms of major depression may turn to alcohol use to relieve their depressive symptoms and as a consequence, a heavy or harmful drinking pattern and alcohol dependence can occur. This phenomenon can, in fact, occur in people of any socio-economic class. However, if those who are in the high class have a better access to alcohol, the likelihood of having a harmful drinking pattern or alcohol dependence could be higher.

On the other hand, people of the lower SES group often face more social difficulties, which enhances susceptibility to negative health outcomes when exposed to risk factors [12, 15, 41]. The higher prevalence of both MDE and AUD among those in the lowest SES group could lend support to this finding. This is consistent with other studies which showed that people who have a low SES and are experiencing a greater social disadvantage generally suffer poor health outcomes, including depressive and substance use disorders, have more disabilities, and poorer access to health care [9, 10, 17, 19, 39, 42, 43]. Whether the strong association between depressive episode and harmful-dependent drinking in the high and low SES groups is driven by a high accessibility to alcohol in the high SES group or an enhanced susceptibility to negative health outcomes when exposed to chronic difficulties among the low SES group is plausible and deserves further research to elucidate mechanisms explaining this social disparity. However, chance cannot be ruled out as an explanation for the observed findings due to the low statistical power as both MDE and AUD are rare in this general population study. Furthermore, the classification of wealth index derived from the PCA might not differentiate people well, thus spurious differences could also be the case [19].

For both groups of AUD, the significant and strong associations with MDE were found only among those completing secondary school education or higher but not among those with primary school education. The reason for this may be similar to the above explanation for the high socio-economic group and the finding further supports the impact of SES on the relationship between the two disorders. Education is known to causally influence health through mechanisms such as creating a greater sense of control, better working conditions, increased social capital, and improved health behaviors [44]. If it is true that highly educated people turn to alcohol when they are depressed to alleviate their dysphoria, it is possible that a depressive condition deforms their sense of control, leading to such poor health behavior and education does not protect against this outcome. This finding highlights the importance of a comprehensive assessment of the co-occurrence of depressive disorder and AUD and providing appropriate treatment and care in all individuals regardless of their education level.

No modifying effect of employment status was found on the association between MDE and AUD. The association was only significant for that between MDE and hazardous or harmful-dependent drinking among the employed group. However, the number of unemployed individuals who were depressed and had either hazardous or harmful drinking was low or none, preventing us from determining the effect of employment status on the relationship between MDE and AUD. The inconsistency of the findings by three SES indicators warrants further studies to explicate mechanisms underlying the socio-economic inequality in the relationship between MDE and AUD. As mentioned in other studies, socio-economic inequalities in health may vary depending on the indicators used to measure SES and no single indicator can provide a full picture of SES of the population [23, 45].

Living in a rural area was found to be protective against harmful-dependent drinking in the univariate analysis and a stronger association was found between MDE and harmful-dependent drinking for people living in urban areas compared to those living in rural areas. Evidence of the relationship between urbanicity with depression and alcohol use varies in the literature [41, 46, 47]. In Thailand, people living in rural areas often have close ties and shared social activities. The poor in these rural areas also live better lives than do those in the urban areas as they have more ready access to food and green space in their surroundings. People living in urban areas can face a lot of hardships related to urban lifestyles, for example, faster pace, higher crime rates, a more crowded environment, limited green space, and higher levels of pollution [48]. Such urban environments may enhance susceptibility to negative health outcomes when exposed to risk factors and increase the risk of poor mental health [49].

Depressive disorders and AUD are common public health problems in Thailand [50] and priority conditions identified in the WHO Mental Health Gap Action Programme [51]. The present findings should be considered in terms of the social context of Thailand where income inequality is in the middle range (GINI coefficient ranges between 37.5 and 39.4 since 2010) [52]. Our findings among the general Thai population suggest that mental health care and promotion would not help to improve the mental health of Thai people to its highest limit should concurrent efforts to reduce social inequalities not be implemented.

Mental health and many mental disorders are shaped by the social, economic, and physical environments where people live [15]. Our findings emphasize the need to implement and scale-up public policies and intervention programmes for depressive and alcohol use disorders among the general population. Evidence-based interventions for depressive disorder include treatment with antidepressants and psychosocial interventions such as cognitive behaviour therapy and problem-solving while those for AUD are policy and legislative interventions including regulation of the availability of alcohol, enactment of appropriate drink-driving policies, reduction of the demand for alcohol through taxation and pricing mechanisms, and interventions for hazardous drinking and treatment of AUD with pharmacological and psychosocial interventions [51]. Our results also suggest that interventions for both conditions should be provided in proportion to the needs of people of different socio-economic groups. Future research is also needed to understand the mechanisms which underlie the different relationships that exist among people of different socio-economic status.

Moreover, the present research should be helpful for clinical practice. In clinical settings where the prevalence of major depression is usually higher than that in the general population, clinicians should also seek for the co-occurrence of AUD, especially harmful-dependent drinking in depressed patients. On the assumption of a causal process of depressive disorder leading to harmful use or alcohol dependence, it can be indicated that some percentage of patients with AUD may actually improve upon treatment of their depressive disorder. There is a number of studies showing the efficacy of psychological interventions among people with co-occurring alcohol use and depressive disorders [53]. This thus suggests that treatment of MDE should include assessment and treatment of AUD or vice versa. The consistent finding across studies that the prevalence of major depression and AUD was high among those with low socio-economic status should be of concern for clinicians. Patients with a lower SES are generally less likely to access medical care whereas they tend to have more disabilities and a poorer prognosis [13, 16], thus they would need more help in a clinical setting. It should also be recognized that for patients who are in a higher SES or education level, or employed, a depressive episode increases their tendency to have alcohol use disorder, thus treatment of both disorders is recommended and should be tailored to their needs.

### Study strengths and limitations

The main strength of this study is the large sample size, nationwide sampling frame, and probability sampling method, making it representative of the general population of the whole country. Furthermore, the possible role of confounding factors such as socio-demographic factors and the presence of chronic medical diseases was taken into account in the analyses. However, there are some limitations that deserve mention. A cross-sectional study is a useful design for obtaining the prevalence of MDE and AUD among the general population, but it cannot establish a causal relationship. The direction of causality is indeterminate; depression and AUD could be reciprocally related to each other by a feedback loop in which drinking increases the risk of depression and the depression leads to an increased consumption of alcohol and related problems [4]. In our study, MDE and AUD were self-reported and the questionnaire used for measuring both conditions was a screening instrument (not a diagnostic instrument), assessing symptoms that occurred in the past 12 months, which may not fit the full criteria of major depressive disorder or alcohol use disorder. Therefore, our results have limited comparability with studies that used diagnostic measures. Furthermore, there were few respondents with MDE who had hazardous or harmful-dependent drinking as the sample was taken from the general population. Although the association between MDE and harmful-dependent drinking was significant across most socio-economic levels, most of the confidence intervals were too wide to make any solid conclusions. Due to the unavailability of data, our study did not take into account other potential confounding factors, e.g. stressful life events and personality profile, which may be associated with both MDE and AUD. Finally, a collider stratification bias might exist but be undetected in our study due to our inability to measure some relevant stratification variables. Inclusion of these variables in the model could strongly mitigate the effect of MDE on AUD. However, it is not likely that the positive association between MDE and AUD found in our study would have been caused by a collider stratification bias as our finding was consistent with the true causality between MDE and AUD established in previous literature [7, 23, 29, 33–38]. In addition, our data came from a national survey sampled from the general population so there is no possibility of collider stratification bias by design.

## Conclusions

This study shows that there is a significant association between MDE and AUD, in particular harmful-dependent drinking. This association is further modified by socio-economic factors such as wealth index, education, and area of residence. Our study further contributes to the growing literature on the social inequalities of mental disorders, which is seen across culture. The varying associations between these two conditions across different socio-economic levels need to be considered when formulating mental health policy for equitable health care and when providing clinical services.

## Data Availability

The data that support the findings of this study are available from the National Health Examination Survey Office, Health Systems Research Institute, Thailand, but restrictions apply to the availability of these data and so are not publicly available. Data are available upon reasonable request and with permission of the investigator committee of the Fifth National Health Examination Survey (Contact: Professor Wichai Aekplakorn, Department of Community Medicine, Faculty of Medicine Ramathibodi Hospital, Bangkok, Thailand: wichai.aek@mahidol.ac.th).

## Abbreviations

AUD: Alcohol use disorder
MDE: Major depressive episode
AOR: Adjusted odds ratio
CI: Confidence intervals
DSM-IV: Diagnostic and statistical manual of mental disorders fourth edition
SES: Socio-economic status
NHES-5: Thailand’s fifth National Health Examination Survey
IHRP: Institute for the Development of Human Research Protection
AUDIT: Alcohol Use Disorder Identification Test
WHO: World Health Organization
MINI: Mini-International Neuropsychiatric Interview
SD: Standard deviation
